# Pigmented purpura dermatosis associated with compounded semaglutide: A case report and literature review of medication-associated pigmented purpuric dermatosis

**DOI:** 10.1016/j.jdcr.2026.08.020

**Published:** 2026-08-21

**Authors:** Stephanie Y. Zhang, Maleka Najmi, Katherine H. Fiala

**Affiliations:** a School of Medicine, Baylor College of Medicine, Houston, Texas; b Department of Dermatology, Baylor Scott & White Memorial Hospital, Temple, Texas

**Keywords:** capillaritis, drug-induced pigmented purpuric dermatoses, general dermatology, pigmented purpuric dermatoses, GLP-1 agonists, Schamberg’s, semaglutide

## Background

The pigmented purpuric dermatoses (PPD) are a group of cutaneous diseases characterized by petechial hemorrhage resulting from capillaritis.^1^ The hallmark pathological features of PPD include extravasation of erythrocytes, hemosiderin deposition in the papillary dermis, perivascular lymphocytic infiltrate centered on superficial capillaries and endothelial cell swelling.^2^ Patients usually present with a non-palpable, purpuric eruption on bilateral lower extremities that may be mistaken for vasculitis or thrombocytopenia.

The pathophysiology of the PPD remains unclear but certain medications have been established in the literature as etiologic factors for PPD.^2^^,^^3^ Medications commonly known to instigate PPD include NSAIDs, diuretics, sedatives and antibiotics.^3^^,^^4^

## Case report

We present a 55-year-old male who was referred to dermatology for evaluation of a persistent, non-blanching rash that was nonpruritic, nonpainful, and nonbleeding. The eruption began approximately 2 weeks after switching to a new formulation of compounded injectable semaglutide. The rash initially appeared within the intergluteal folds and subsequently spread around the pelvis and extended down the bilateral thighs (Fig 1, *A* and *B*). The patient had previously been treated with compounded semaglutide for 1 year for weight loss management without adverse effects when the compounding pharmacy was changed due to cost. Both formulations included Vitamin B6 to assist with the risk of nausea. The patient reported onset of the rash soon after the first injection of the new formulation with continued worsening for the following 4 weeks of injections. He denied any prior history of intense exercise or prolonged travel. At this time, he was counseled to discontinue the medication. He then used topical triamcinolone 0.1% ointment twice daily for 3 months.

**Fig 1:**
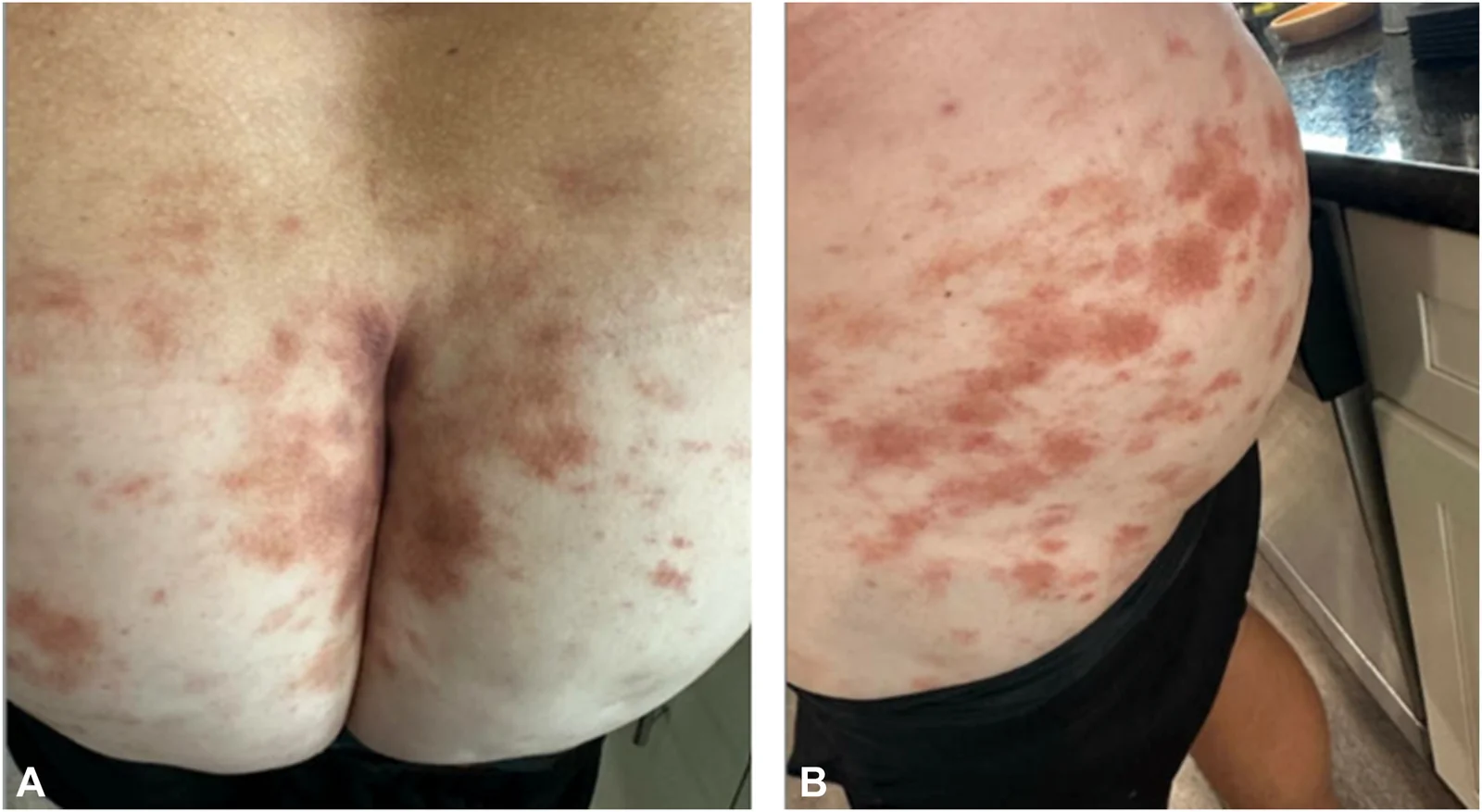
**A,** Initial non-blanching, “cayenne pepper-like” macules and patches in intergluteal folds and scattered along bilateral gluteal regions. **B,** Left hip with scattered red macules and patches, similar to appearance on the right hip (not featured).

The differential included PPD, PPD-like mycosis fungoides, small vessel cutaneous vasculitis and possibly symmetrical drug-related intertriginous and flexural exanthema (SDRIFE). A laboratory evaluation was performed to assess for potential systemic etiologies. A comprehensive metabolic panel, erythrocyte sedimentation rate, C-reactive protein, complement levels, antinuclear antibody, indirect immunofluorescence assay, rheumatoid factor, antineutrophil cytoplasmic antibodies, hepatitis C antibody, and hepatitis B surface antibodies were obtained and all within normal limits. Due to persistence of the eruption, a shave biopsy was obtained from skin of the left hip. Histopathologic examination demonstrated a superficial perivascular lymphocytic infiltrate with prominent red blood cell extravasation and hemosiderin-laden macrophages, findings consistent with PPD.

The patient had been off all forms of semaglutide for 3 months when he was restarted on his original formulation for weight loss management. At his dermatology clinic, the rash had improved significantly after being on the original compounded formulation for 2 months. Physical examination revealed brown stippled macules within the intergluteal folds consistent with hemosiderin deposition and golden-brown stippled pigmentation on the bilateral hips; the previously noted erythema had resolved (Fig 2, *A* and *B*). Among the clinical variants of PPD, Shamberg disease was favored given the asymptomatic presentation. The patient was advised to initiate rutoside 50 mg twice daily and vitamin C 500 mg daily for 6 months for their proposed vasoprotective effects.

**Fig 2:**
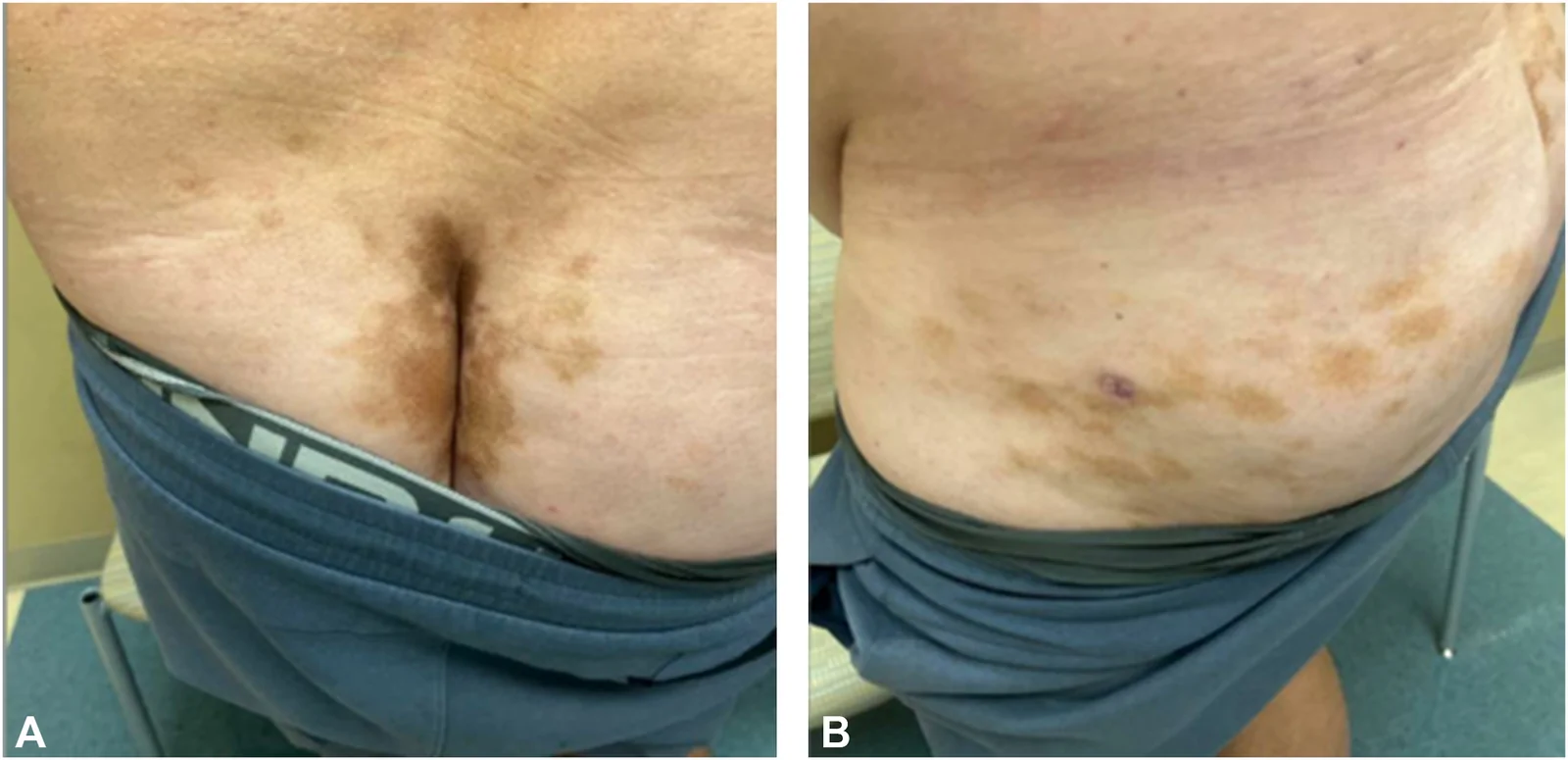
**A,** Brown stippled macules on intergluteal folds 5 months after initial presentation. **B,** Left hip with residual golden-brown stippled macules, consistent with iron/hemosiderin, right hip with similar appearance (not featured).

## Discussion

PPD results from minimal inflammation and hemorrhage of superficial papillary dermal vessels, usually capillaries. The cause for inflammation is unknown with no association with coagulation abnormalities.^2^ Studies have suggested that this is likely a CD8+ T-cell mediated process given findings of CD8+ T-cell predominance in histologic lymphocytic infiltrates.^5^^,^^6^ Each clinical variant has characteristic clinical features and associated demographic distribution. Schamberg disease commonly occurs in middle-aged and older men.^1^^,^^2^ Clinical exam usually shows “cayenne pepper” petechiae that will flow into irregularly-outlined oval yellow-orange-brown patches with classical involvement of the lower limbs.^2^ The majority of patients are asymptomatic as in our case.

Although the exact pathophysiology of PPD remains unclear, several contributing factors have been cited in the literature, including physical activity, venous insufficiency, capillary fragility, hypertension, contact allergy to dyes, various chemical exposures and medications.^1^^,^^7^ It is theorized that in drug-induced instances of PPD, the substance acts as a hapten that forms antigens with human proteins that stimulate antibody formation and result in immune-mediated vascular injury.^2^^,^^3^ Direct immunofluorescence studies have supported this immune complex deposition theory by showing vascular deposition of either immunoglobulin, complement or fibrinogen in 82% of studied cases.^8^ As a result, clinically, drug-induced cases are more likely to be generalized but still present with classic lower-extremity involvement.^4^ Involvement of atypical sites including the buttocks, trunk and upper extremities greatly favors a drug etiology since idiopathic PPD overwhelmingly favors the lower extremities (96.3%).^2^ To our knowledge, our case represents the first drug-induced PPD with a buttock-predominant distribution and in association with compounded semaglutide.

A review of the literature reveals a wide variety of substances and medications that have been cited to provoke PPD. Diuretics and analgesics are common culprits.^3^^,^^4^ However, we have encountered no reports of semaglutide, a GLP-1 agonist used in type 2 diabetes and chronic weight management, causing PPD.

Interestingly, in our patient, the lesions did not appear until after the patient switched the formulation of his semaglutide. While other cases of drug-induced PPD occurred after months to years of drug consumption, in our patient, the rash appeared within 2 weeks of drug initiation and faded with discontinuation of the compounded semaglutide.^3^ Both the time course and pathology results are consistent with previously described cases of drug-induced PPD.^4^ One hypothesis is that the new formulation had contaminants that served as a trigger. Compounded medications are not FDA approved and one of the well-known associated risks is contamination.^9^

Management of Schamberg’s includes topical steroids for inflammation, oral rutoside, vitamin C and occasionally pentoxifylline to improve blood flow.^1^ In drug-induced cases, removal of the offending medication is often first line. Our case raises an important clinical consideration. Drug-induced PPD does not always require the patient to start a new medication; altered formulation of a preexisting medication may be the cause. GLP-1 agonists are increasingly prescribed by primary care physicians and in dermatology due to their beneficial effects in inflammatory conditions like hidradenitis suppurativa and psoriasis.^10^ Thus, it is important when obtaining a history to ask about medication formulations.

## Conflicts of interest

None disclosed.
